# Data-Driven Sparse Reconstruction Framework for Anomaly Detection in Smart Manufacturing Environment

**DOI:** 10.3390/s26206523

**Published:** 2026-10-14

**Authors:** Jung Kyu Park, Youngmi Baek

**Affiliations:** Department of Computer Engineering, Daejin University, Pocheon-si 11159, Republic of Korea; jkpark@daejin.ac.kr

**Keywords:** anomaly detection, sensor reliability, sparse linear inverse problem, effective sensing matrix

## Abstract

Reliable anomaly detection of sensor measurements is essential for monitoring complex systems in smart manufacturing environments. However, conventional approaches often require predefined statistical assumptions or explicit knowledge of physical measurement models. Therefore, a data-driven sparse reconstruction framework (DSRF) is proposed for anomaly detection without requiring explicit noise distribution or separate identification of a physical measurement model. The proposed framework decomposes observation deviations into a reconstructed normal measurement component, a sparse anomaly residual, and a remaining reconstruction residual. The DSRF is evaluated using current and vibration measurements from robot welding systems. Normal measurements are divided into training, validation, and independent test subsets, while labeled abnormal samples are reserved for final evaluation. Training data are used to learn an effective sensing matrix and determine anomaly thresholds. At the 99th-percentile threshold, DSRF achieves 100% detection with no false negatives and accuracies of 99.76% and 98.38% for the current and vibration datasets, respectively. Comparative experiments with PCA reconstruction and an autoencoder show similarly high classification performance while DSRF only provides a larger threshold-normalized separation and explicitly estimates sparse anomaly residuals. The anomaly residual also provides frequency feature-level information for interpreting prominent abnormal deviations. These results demonstrate the feasibility of DSRF for anomaly detection and residual-based interpretation without explicit noise assumptions or separately identified physical system models.

## 1. Introduction

In smart manufacturing environments, predictive maintenance represents a fundamental technological challenge in enabling data-driven intelligent factories [1]. It is a method that analyzes various sensor data collected from a given equipment to identify early signs of abnormal conditions and enables maintenance actions to be taken before a failure happens. Compared to traditional reactive maintenance, it reduces equipment downtime and helps lower costs while improving overall productivity. Predictive maintenance is particularly critical for industrial equipment. Electric motors are a key representative component in industrial equipment due to their widespread use and operational importance [2,3]. In addition, most automation and manufacturing processes depend on them for reliable operations. For improving motor reliability and reducing maintenance costs, monitoring the condition of bearings, which are critical mechanical components in electric motors, is essential. This is because it is known that approximately 60% of motor failures in industrial environments are caused by bearing damage [4]. In this regard, sensor fault detection systems can detect bearing abnormalities at an early stage by utilizing data collected from various sensors. However, in spatially constrained environments such as bearing systems, installing multiple sensors is challenging. As multiple sensors mounted on the equipment measure different physical variables such as vibration and current, these measurements can be utilized as condition monitoring variables to evaluate the condition of the equipment.

Sensor faults can manifest in various forms including bias errors, stuck-at faults, drift errors, data loss, noise, and temporary faults (i.e., transient faults), which can lead to abnormal sensor measurements [5,6]. Among them, stuck-at faults and data loss can be identified immediately. In contrast, bias errors, drift errors, and transient faults require analysis of temporal trends for accurate detection. In particular, sensors may experience transient faults which potentially lead to temporary malfunctions and irregular measurements. In contrast to permanent faults, transient faults do not persist over time and allow the sensor to eventually return to normal condition. However, if transient faults occur repeatedly over time, they may indicate underlying sensor or machine degradation, component aging, or changes in the operating environments. Furthermore, in industrial automation systems, even short-duration errors caused by incorrect measurement values can affect control and lead to abnormal system operations. In recent smart manufacturing systems, as a cyber–physical system, transient faults may also be intentionally induced by malicious attackers who may manipulate sensor measurements so that the sensor behaves as if it were experiencing a transient fault [7,8]. Whether caused by natural sensor malfunctions or malicious attacks, transient faults represent temporary deviations from normal sensor behavior. These deviations may lead to inaccurate condition assessment and unreliable fault prediction, while it is often difficult to detect them due to their short duration. This study considers transient faults among sensor faults as a type of anomaly because they indicate abnormal behavior in sensor measurements. Therefore, in this paper, anomalies are defined as deviations from normal sensor observations or operating behaviors, including various types of sensor faults in smart manufacturing environments.

To effectively and accurately identify such anomalies from sensors, several approaches have been developed, including sensor fusion, analytical redundancy, statistical fault detection, and machine learning-based anomaly detection [6]. These technologies have several limitations. For instance, a sensor fusion method for detecting anomalies relies on multiple sensors to measure the same physical quantity, which leads to increased device and maintenance costs. In addition, they may produce different outputs due to variations in measurement locations, sensor characteristics, and accuracy [9]. Moreover, if multiple sensors fail simultaneously due to the same environmental factors, the method cannot effectively identify anomalies.

The analytical redundancy approach is based on exploiting correlations among different sensors. However, in real manufacturing environments, these correlations may vary significantly even under normal operation due to changes in load conditions, lubrication states, and temperature [10]. As a result, the reliability of the correlation-based models may decrease, and developing robust modes that consider various operating conditions becomes more complex and time-consuming [11].

Since statistical detection methods rely heavily on predefined thresholds, their detection performance and sensitivity can vary significantly according to selected threshold values [11,12]. Their performance may also deteriorate when vibration data do not derive from a normal distribution or are affected by noise [3,11]. Moreover, this statistical method may have limitations in detecting gradual drifts or early-state degradation as their characteristics can be similar to normal variations caused by changing operating conditions and measurement noise.

Machine learning-based detection methods are affected by data imbalance, as normal operating data are plentiful whereas actual fault data are sparse. Additionally, changes in environmental conditions may degrade the generalization performance of the trained model because the characteristics of the sensor data differ from those observed during training [13]. This often results in frequent false positives and false negatives [11,13]. Moreover, the opacity of machine learning-based methods makes it challenging to explain their decision-making processes and to establish reliability in the decision system.

Nevertheless, to improve the reliability and effectiveness of predictive maintenance, developing an explainable model tailored to the characteristics of a specific process is more important than pursuing a generally applicable model [14,15]. In particular, it is important to assess the reliability of the observed data together with anomaly detection since the performance of any anomaly detection model depends strongly on the sensor measurements. Therefore, this study focuses on enabling anomaly detection and feature-level interpretation within a unified and explainable framework.

Recent deep learning-based intelligent diagnostic methods have also been developed to address limited fault samples, distribution shifts, and varying operating conditions. Representative approaches include conditional distribution-guided adversarial transfer learning networks, adaptive fused domain-cycling variational generative adversarial networks, and dynamic collaborative adversarial domain adaptation networks [16,17,18]. These approaches improve diagnostic performance through nonlinear feature learning, generative modeling, adversarial transfer learning, and domain adaptation under challenging operating conditions. Nevertheless, interpretability and explainability remain important considerations when data-driven models are applied to predictive maintenance and industrial decision-making tasks [14,15]. Therefore, this study focuses on enabling anomaly detection and feature-level interpretation within a unified framework.

In this paper, we propose a data-driven anomaly detection framework based on the sparse linear inverse problem (SLIP). The proposed framework explicitly decomposes each sensor observation into a low-dimensional structural component explained by the normal measurement structure and a sparse anomaly residual component associated with anomalous deviations that cannot be sufficiently explained by the learned normal structure. The magnitude of the anomaly residual is used for sample-level anomaly detection, while its distribution across measurement features provides supplementary information for feature-level interpretation.

The main contributions of this paper are summarized as follows. First, sensor anomaly detection is formulated as a data-driven sparse linear inverse problem. The observed sensor deviation is decomposed into a component explained by the learned normal measurement structure and a sparse anomaly component. Rather than separately identifying the physical measurement matrix and the underlying state representation, the proposed framework directly constructs and refines an effective sensing matrix from normal sensor measurements. The contribution therefore lies not in introducing a new dimensionality reduction or sparse optimization technique individually, but in integrating these elements within a unified sparse inverse formulation for anomaly detection.

Second, an approximately sparse deviation model is introduced to jointly represent the normal component, sparse anomaly residual, and remaining reconstruction residual. This formulation does not require a predefined statistical noise distribution and allows small modeling discrepancies and measurement variations to remain in the reconstruction residual, while relatively concentrated abnormal deviations are represented through the sparse anomaly residual. This formulation naturally captures normal measurement variability through the reconstruction residual while it preserves sparsity for anomaly detection.

Third, a unified framework for explainable anomaly detection is developed. By jointly estimating the sparse normal representation coefficients and sparse anomaly residuals within the sparse linear inverse formulation, the proposed framework separates the measurement components represented by the learned normal structure from relatively concentrated unexplained deviations. The magnitude of the estimated anomaly residual is used to calculate the sample-level anomaly score while its distribution across measurement features provides a basis for frequency feature-level interpretation of prominent abnormal deviations. This enables anomaly detection and feature-level interpretation within the same sparse inverse framework without requiring an additional classification model.

The remainder of this paper is organized as follows. Section 2 presents the theoretical background and considerations underlying the proposed methodology. Section 3 describes the proposed data-driven sparse reconstruction framework that consists of constructing observation deviations from normal operating measurements, learning the effective sensing matrix, jointly separating the reconstructed normal measurement component from the sparse anomaly residual, and calculating the anomaly score. Section 4 provides the experimental setup, evaluation, and experimental results using current and vibration measurements collected from industrial equipment. Finally, Section 5 concludes this paper and presents directions for future work.

## 2. Background and Considerations for Our Methodology

### 2.1. Linear Inverse Problem in Various Sensors

In a linear system, where y denotes the measurements obtained from sensors and A is a known linear operator, the problem of estimating the unknown state vector (i.e., original data) x from the observed measurements is referred to as a linear inverse problem [19,20]. A general linear inverse problem is formulated as follows:

(1) y=Ax+v

where $y\in R^{m}$ is the observed vector with measurement dimension m, $x\in R^{n}$ represents the true state vector with state dimension n, $A\in R^{m\times n}$ is the measurement matrix, and $v\in R^{m}$ denotes the additive noise vector. The observed vector y corresponds to measurements collected under normal operating conditions. The measurement matrix A is a linear transformation matrix which linearly maps the true state vector x from the original state space to the observation space. In many sensor-based systems, m denotes the number of sensor measurements while n denotes the number of state variables to be estimated. The state variables characterize the underlying physical state of the given system. Moreover, the additive noise vector v represents inherent sensor uncertainty, environmental disturbances, and other measurement variations occurring under normal operating conditions.

To facilitate an intuitive understanding of the linear inverse problem, a simplified example is introduced in this paper. Each sensor is assumed to provide a single measurement. Consequently, the number of sensor measurements m is equal to the number of sensors. Also, it is assumed that the number of sensors is equal to the number of state variables n for simplicity. This square system is adopted solely for illustrative purposes and the proposed approach is not limited to the case of m=n. In this example, four sensors and four state variables are considered.

The observed vector y is constructed from the observed data obtained from these four sensors and can be expressed as follows.

(2) $$\left[\begin{array}{c} \begin{array}{c} y_{1}\\ y_{2}\\ y_{3} \end{array}\\ y_{4} \end{array}\right]=\left[\begin{array}{c} \begin{array}{c} a_{11}\\ a_{21}\\ a_{31} \end{array}\\ a_{41} \end{array}\;\begin{array}{c} \begin{array}{c} a_{12}\\ a_{22}\\ a_{32} \end{array}\\ a_{42} \end{array}\;\begin{array}{c} \begin{array}{c} a_{13}\\ a_{23}\\ a_{33} \end{array}\\ a_{43} \end{array}\;\begin{array}{c} \begin{array}{c} a_{14}\\ a_{24}\\ a_{34} \end{array}\\ a_{44} \end{array}\right]\left[\begin{array}{c} \begin{array}{c} x_{1}\\ x_{2}\\ x_{3} \end{array}\\ x_{4} \end{array}\right]+\left[\begin{array}{c} \begin{array}{c} v_{1}\\ v_{2}\\ v_{3} \end{array}\\ v_{4} \end{array}\right]\;\;$$

When x is represented as a four-dimensional state vector, the measurement matrix A combines the four components of x to produce the four observed sensor measurements y. The state vector x represents the true state of the system and consists of four state variables $x_{i}$, which correspond to internal physical variables characterizing the operating conditions of the equipment. The sensor measurements y are generated as linear combinations of components of x through the measurement matrix $A\in R^{4\times 4}$. In this regard, each row of A contains the linear weights that map the state variables to a single sensor measurement while each column of A represents the influence of a particular state variable on the sensor measurements. Therefore, each element $a_{ij}$ of the measurement matrix A represents the contribution of each state variable $x_{j}$ to the sensor measurement $y_{i}$ (1≤i,j≤4). Since the state variables in x are not directly observable, state variables x can, in principle, be indirectly estimated from the observed sensor measurements y by solving the corresponding inverse problem, under the assumption that the measurement matrix A contains sufficient information for state estimation.

### 2.2. Data-Driven Sparse Linear Inverse Formulation with Inherent Measurement Variability

The sparse linear inverse problem (SLIP) extends the conventional linear inverse problem by introducing a sparsity constraint on the unknown state [21]. In other words, the state vector $x\in R^{n}$ is assumed to admit a sparse representation over a dictionary matrix $D\in R^{n\times K}$ as follows:

(3) x=Dα

where $\alpha \in R^{K}$ denotes a sparse coefficient vector. The SLIP aims to recover a sparse coefficient vector α from linear measurements in both the absence and presence of measurement noise.

In the context of anomaly detection, the measurement noise may be introduced to account for inherent sensor uncertainty and normal measurement variability. We focus on the limitation of the model assumption that may arise when the actual noise characteristics deviate from a predefined noise distribution.

In many statistical and Bayesian inverse-problem formulations, measurement uncertainty is represented by a separate additive noise term characterized by an assumed statistical model. However, model misspecification may adversely affect the reliability of the inferred solution when the actual measurement uncertainty deviates from the assumed noise distribution [22,23]. Therefore, this study does not impose a predefined probability distribution on measurement noise. It is assumed that learning directly from actual normal sensor measurements enables the construction of a data-driven state representation that implicitly captures the statistical characteristics of normal measurement variability, including sensor uncertainty and environmental variations, without assuming a predefined probability distribution for measurement noise.

In this regard, the conventional state vector x is replaced by a data-driven effective state vector $\tilde{x}$ which reflects the variability embedded in actual normal sensor measurements under normal operating conditions. Therefore, the normal measurement variability previously represented by the additive noise term v is implicitly incorporated into the data-driven effective state vector $\tilde{x}$. In other words, it reflects the statistical characteristics of actual normal measurements, including the combined effects of sensor uncertainty, environmental variations, and normal operating variability. These sources of variability are neither separately decomposed nor modeled using predefined probability distributions. Instead, their statistical characteristics are implicitly captured through the state representation learned from actual normal measurements.

Accordingly, the linear observation model is expressed as follows:

(4) $$y=A\tilde{x}$$

Unlike the conventional state vector x, the effective state vector $\tilde{x}$ does not represent an ideal noise-free physical state but the data-driven effective state reflects the variability present in actual measurements.

Under the data-driven SLIP formulation, each observed measurement $y_{i}$ is a linear combination of all elements of the effective state vector $\tilde{x}$, where the weights are given by the corresponding row of the measurement matrix A. The effective state vector under normal operating conditions is further assumed to admit a sparse representation over a dictionary matrix D:

(5) $$\tilde{x}=D\alpha$$

Substituting Equation (5) into Equation (4) yields

(6) y=ADα

The objective of the data-driven SLIP formulation is to recover the sparse coefficient vector α from the observed measurements. In the theoretical formulation, the effective state deviation is assumed to admit a sparse representation over the dictionary matrix D. However, in the practical implementation considered in this study, the effective state variables and the dictionary matrix D are not separately identified. Since only sensor observations are available and no additional information is provided to uniquely identify the physical measurement matrix A and the dictionary matrix D, these two matrices cannot generally be separately identified. Therefore, their combined effect is represented by an effective sensing matrix which is constructed directly from normal sensor measurements in the observation space, as described in Section 3.

In the theoretical representation, the columns of the dictionary matrix D represent structural patterns of the effective state while the sparse coefficient vector α specifies the combination of these patterns used to represent a particular effective state deviation. In the practical implementation, this combined mapping represented theoretically by A and D is handled through the effective sensing matrix, and the corresponding sparse coefficients are estimated directly in the observation space.

In this manner, the data-driven formulation incorporates the statistical characteristics of normal measurement variability while it preserves the sparse structures of normal operating conditions.

### 2.3. Sparse Linear Inverse Problem Formulation of Sensor Data for Anomaly Detection

When an equipment system operates normally, most state variables $x_{i}$ maintain stable values and do not undergo significant changes. In other words, under a given steady operating condition, the effective state variables ${\tilde{x}}_{i}$ generally fluctuate within a limited range around their reference values. The deviation vector $\Delta \tilde{x}$ is defined as

(7) $$\Delta \tilde{x}=\tilde{x}-{\tilde{x}}_{ref},$$

where ${\tilde{x}}_{ref}$ denotes the reference effective state learned from normal operating measurements. Under normal operating conditions, only a small subset of the effective state variables is expected to exhibit noticeable deviations from the reference effective state while the remaining variables stay close to their reference values. It should be noted that the reference effective state ${\tilde{x}}_{ref}$ represents the expected effective state under normal and non-fault operating conditions and reflects the inherent variability present in actual measurements. Therefore, the deviation vector $\Delta \tilde{x}$ can be regarded as approximately sparse under normal operating conditions since only a small number of state variables exhibit noticeable deviations while the majority remain close to their reference values.

For instance, we assume that the effective state vector $\tilde{x}$ consists of rotational speed (rotations per minute), current, vibration, and acoustic noise level. Under a steady normal operating condition, each effective state variable remains close to its corresponding reference value. Therefore, the corresponding deviation remains close to zero. This simplified example assumes that each component of the effective state vector corresponds to one dominant physical variable even though actual sensor measurements may generally be influenced by multiple state variables. In this case, the deviation vector $\Delta \tilde{x}$ exhibits an approximately sparse structure because only small deviations from the reference effective state ${\tilde{x}}_{ref}$ are observed under steady normal operating conditions, as illustrated inEquation (8). Although the deviation components are not exactly zero because the actual measurements contain inherent variability, their magnitudes remain small under steady normal operating conditions.

(8) $$\Delta \tilde{x}=\left[\begin{array}{c} \begin{array}{c} {∆\tilde{x}}_{1}\\ ∆{\tilde{x}}_{2}\\ {∆\tilde{x}}_{3} \end{array}\\ {∆\tilde{x}}_{4} \end{array}\right]=\left[\begin{array}{c} \begin{array}{c} 0.02\\ -0.01\\ 0.03 \end{array}\\ 0.01 \end{array}\right]$$

On the other hand, when an abnormal condition such as a bearing defect occurs, one or a few deviation components related to a specific variable, such as vibration, may increase significantly in magnitude. Therefore, the deviation vector $\Delta \tilde{x}$ becomes approximately sparse, with only a certain component exhibiting large-magnitude deviation, as illustrated in Equation (9). Even under abnormal operating conditions, the deviation vector often remains approximately sparse because fault-related deviations are typically localized to a limited number of effective state variables. However, the pattern (i.e., locations and magnitudes) of activated components differs from those observed under steady normal operating conditions.

(9) $$\Delta \tilde{x}=\left[\begin{array}{c} \begin{array}{c} {∆\tilde{x}}_{1}\\ ∆{\tilde{x}}_{2}\\ {∆\tilde{x}}_{3} \end{array}\\ {∆\tilde{x}}_{4} \end{array}\right]=\left[\begin{array}{c} \begin{array}{c} 0.03\\ 0.04\\ 2.80 \end{array}\\ 0.05 \end{array}\right]$$

To exploit this approximate sparsity with near zero, the deviation vector $\Delta \tilde{x}$ is represented using the dictionary matrix D with a sparse coefficient vector α, as follows:

(10) $$\Delta \tilde{x}=D\alpha .$$

Assuming that both the given and reference effective states are mapped to the observation space through the same measurement matrix A, the observation deviation Δy in the observed measurements is expressed as

(11) $$\Delta y=A\Delta \tilde{x},$$

where Δy is equal to the difference between the observed vector and the reference observation vector (i.e., $\Delta y=y-y_{ref}$). Substituting the sparse representation in Equation (10) into Equation (11) yields

(12) $$\Delta y=A\Delta \tilde{x}=AD\alpha =\Phi \alpha$$

where Φ denotes the effective sensing matrix and is defined as Φ=AD.

Consequently, in this paper, the SLIP is reformulated as the recovery of the sparse coefficient vector α from the observation deviation Δy through the effective sensing matrix Φ. The columns of the dictionary matrix D represent the structural patterns of the effective state. In this theoretical representation, only a small subset of the columns of the dictionary matrix is required to represent an approximately sparse effective state deviation because the deviation vector $\Delta \tilde{x}$ is approximately sparse: only a limited number of effective state deviations are significant. This sparse representation reflects the intrinsic low-dimensional structure of normal operating conditions. The measurement matrix A describes the linear sensing process that projects deviations $\Delta \tilde{x}$ in the effective state variables into deviations Δy in the sensor observations. Combining the measurement matrix A with the dictionary matrix D yields the effective sensing matrix Φ, which directly relates the sparse coefficient vector α to the observed observation deviation Δy.

Under normal operating conditions, the sparse coefficient vector α can be estimated by minimizing the reconstruction error between the observation deviation Δy and the reconstructed observation deviation Φα. Consequently, the reconstruction process accurately represents Δy which results in a small reconstruction error. However, abnormal observations may contain patterns that cannot be adequately represented by the effective sensing matrix constructed from normal operating data. Consequently, it results in an increased reconstruction error since the sparse representation generally becomes less accurate. This enables discrimination between the normal and abnormal operating states based on sparse reconstruction errors rather than direct thresholding of individual sensor measurements.

Therefore, in equipment condition monitoring, the dictionary matrix D captures the intrinsic low-dimensional structure of normal operating conditions while the measurement matrix A models the linear sensor observation process that maps effective state deviations $\Delta \tilde{x}$ to sensor observation deviations Δy. Their combination forms the effective sensing matrix Φ which enables anomaly detection through sparse linear inverse modeling in a computationally efficient manner.

The decomposition Φ=AD provides a conceptual interpretation that separates A, representing the physical sensing process, from D, representing the structural representation of the effective state. In practical implementation, however, A and D may not be separately identifiable when the effective state variables are not directly observed. In such cases, the effective sensing matrix Φ can be learned directly from normal sensor measurements, as described in Section 3.

### 2.4. Methodological Differences from Existing Approaches

Although the proposed data-driven sparse linear inverse model effectively captures the intrinsic structure of normal operating conditions, actual sensor measurements may include abnormal components caused by sensor faults, cyber-attacks or unexpected operating conditions. Since such abnormal components cannot be sufficiently represented by the normal measurement structure characterized by the effective sensing matrix, they produce increased unexplained components. For this reason, the proposed methodology explicitly decomposes the observation deviation into a normal structural component and an abnormal residual component through sparse inverse modeling.

Specifically, the observation deviation is decomposed into a normal component represented through the effective sensing matrix and an anomaly residual component. The normal component is reconstructed through sparse coefficients over the learned dictionary, while the residual represents measurement patterns that cannot be explained by the normal operating model. By jointly estimating the sparse representation of the normal component and the anomaly residual, the proposed framework simultaneously reconstructs the normal measurement component while it isolates unexplained deviations. Consequently, the anomaly residual provides an indicator of inconsistency with the learned normal measurement structure and can be used as the anomaly score.

Recent dictionary learning-based anomaly detection methods mainly detect anomalies by learning a sparse representation of normal observations directly from the measured data and measuring reconstruction errors [23,24,25]. In contrast, the proposed framework introduces a sparse linear inverse interpretation in which the observation deviation is modeled through an effective sensing matrix. Theoretical decomposition of the effective sensing matrix into the measurement matrix A and the dictionary matrix D provides a conceptual interpretation of the sensing process and the structural representation while the practical implementation operates directly in the observation space using the constructed effective sensing matrix. Therefore, the proposed method does not require separate estimation of the physical measurement matrix, dictionary matrix, or effective state variables. The distinctive methodological contributions of this study compared with the existing studies are summarized as follows. First, the existing selective dictionary learning method focuses on learning a discriminative dictionary to improve the reconstruction of normal samples while reducing the representation capability for abnormal observations [23]. In other words, the dictionary indirectly performs anomaly discrimination by the value of the reconstruction error. However, it operates directly in the observation space and does not formulate the relationship between normal structural representation and sensor observations within a sparse linear inverse framework.

In this paper, we do not primarily aim to learn a discriminative dictionary. We formulate equipment condition monitoring as a data-driven SLIP by providing an inverse-problem interpretation of the relationship between the normal structural representation and the sensor observation space.

Second, the existing dictionary learning with one-class SVM (Support Vector Machine) enhances anomaly discrimination by applying an additional one-class SVM classifier after sparse feature extraction [24]. However, anomaly detection relies on a separate classification stage rather than directly estimating the underlying system state or abnormal residuals. We do not rely on a separate classifier by jointly estimating the effective state through sparse reconstruction and identifying abnormal conditions using sparse residual estimation. Furthermore, the proposed formulation provides both sample-level anomaly detection and feature-level interpretation of prominent deviations from the learned normal structure.

Third, the existing dictionary learning with cone atoms enhances the robustness of dictionary learning by constructing cone-shaped dictionary atoms for anomaly detection [25]. In other words, constructing cone-shaped dictionary atoms helps capture inherent variations in normal operating data. In addition, their study focuses on dictionary optimization and does not explicitly model the relationship between the physical state and sensor observations. We focus on a data-driven SLIP formulation that conceptually relates the effective state representation to the sensor observation space through the effective sensing matrix, rather than on dictionary design itself. Furthermore, we model the deviation in the effective state as an approximately sparse vector and jointly estimate the normal-state representation and sparse abnormal components represented by anomaly residuals.

Recent studies have addressed industrial fault diagnosis and anomaly monitoring from several complementary perspectives. An unsupervised hybrid-variable monitoring framework is proposed to integrate binary and continuous process variables, while a ParallelGraphNet-based multi-sensor optimization method is developed for high-speed elevator fault diagnosis [26,27]. These studies demonstrate the effectiveness of system-level monitoring and the exploitation of heterogeneous or multi-sensor information. An integrated sensor anomaly detection and reconstruction framework based on PCA (Principal Component Analysis) has also been proposed for nuclear power plants [28]. This approach is particularly relevant to the present study because it also employs PCA for sensor anomaly detection and reconstruction. However, in this study, we do not use PCA reconstruction error directly as the anomaly indicator. Instead, PCA is used as an initial representation of the normal measurement structure, after which the proposed sparse inverse formulation explicitly separates the normal measurement component from the anomaly-related component.

Furthermore, recent studies have also focused on challenging diagnostic conditions such as small-sample learning, unseen fault categories, weak faults, and real-time monitoring. A dynamic graph meta-learning framework is introduced. It exploits multi-sensor spatial dependencies for cross-category small-sample fault diagnosis [29]. To address unseen fault categories under limited fault samples, a knowledge–data synergy framework for zero-shot composite fault diagnosis is proposed [30]. To address the difficulty of diagnosing early weak faults under strong background noise and complex operating conditions, an optimized recurrence-plot method combined with an improved ResNet is developed [31]. In addition, there is a real-time VMD-CNN-DANet framework for aero-engine bearing fault diagnosis using multi-sensor signals [32]. A multi-scale dynamic graph state space model is proposed for multivariate telemetry anomaly detection [33]. These approaches mainly improve diagnostic performance by learning nonlinear, temporal, multi-sensor, or cross-category representations. In contrast, we focus on explicitly decomposing the observation deviation into a reconstructed normal measurement component, a sparse anomaly residual, and a remaining reconstruction residual. Therefore, our contribution is not an additional classification architecture or a new use of PCA itself, but a data-driven sparse inverse decomposition that directly separates anomaly-related measurement components from the normal measurement structure.

The key novelty of this study is not the use of sparse representation itself, but the reformulation of anomaly detection as a data-driven sparse linear inverse problem based on an effective state representation.

## 3. Data-Driven Sparse Reconstruction Framework for Anomaly Detection Sensors

This section presents the overall framework of the proposed data-driven sparse reconstruction framework for sensor anomaly detection.

In this paper, we propose a novel data-driven sparse reconstruction framework (DSRF) for reliable anomaly detection in smart manufacturing environments. The proposed DSRF consists of four major stages. First, sensor measurements collected under normal operating conditions are used to construct the observation deviation matrix and to construct and refine an effective sensing matrix Φ that represents the mapping relationship between sparse normal-state structures and sensor observation deviations. Second, the observation deviation vector Δy is formulated as a data-driven sparse linear inverse problem using the effective sensing matrix learned directly from normal sensor measurements. Third, the proposed DSRF jointly estimates the sparse coefficient vector α for reconstructing the normal measurement component of the observation deviation and the sparse abnormal residual component, modeled as the sparse anomaly residual. Finally, the estimated sparse anomaly residual is used to calculate the anomaly score and to provide supplementary feature-level information about prominent abnormal deviations.

As described in Section 2.3, the effective state vector $\tilde{x}$ provides a conceptual data-driven representation that incorporates the statistical characteristics of actual sensor measurements that include sensor uncertainty, environmental variations, and normal operating variability. Conceptually, the deviation vector of the effective state $\Delta \tilde{x}$ from its reference state is defined in Equation (7) as $\Delta \tilde{x}=\tilde{x}-{\tilde{x}}_{ref}$. In the theoretical formulation, the effective state deviation $\Delta \tilde{x}$ is assumed to admit an approximately sparse representation over the dictionary matrix ***D***, as defined in Equation (10). Since the effective state deviation $\Delta \tilde{x}$ is mapped to the sensor observation space through the measurement matrix A representing the physical measurement process, the corresponding observation deviation is expressed as $\Delta y=A\Delta \tilde{x}=AD\alpha$ = Φα, where Φ=AD denotes the effective sensing matrix.

Under practical smart manufacturing environments, abnormal patterns representing anomalies tend to occur occasionally and locally in a subset of effective state variables, at specific time instances, or within a limited number of extracted features rather than affecting the entire observed vector. Therefore, these localized abnormal components are modeled as a sparse anomaly residual vector e within the sparse linear inverse formulation. To account for these localized abnormal components, the observation deviation model Δy is extended as

(13) $$\Delta y=A\Delta \tilde{x}+e+\eta$$

where e denotes a sparse anomaly residual and η represents small modeling errors and unmodeled measurement uncertainties.

Substituting the sparse representation in Equations (10) and (12) into the observation deviation model in Equation (13) yields

(14) Δy=Φα+e+η,Φ=AD,

where Φ denotes the effective sensing matrix. Although the effective sensing matrix is theoretically expressed as Φ=AD, neither the physical measurement matrix A nor the corresponding effective state variables are directly available in the sensor datasets used in this study. Therefore, the measurement matrix A and the dictionary matrix D cannot be identified separately in the practical implementation. Instead, their combined effect, represented by the effective sensing matrix Φ, is learned directly from sensor measurements collected under normal operating conditions. Accordingly, the subsequent optimization is performed in the observation space using the learned effective sensing matrix.

The normal measurement component Φα represents the observation deviation explained by the learned normal measurement structure while the sparse anomaly residual vector e represents measurement patterns that cannot be sufficiently explained by the learned normal model. Such unexplained patterns may be caused by sensor faults, abnormal measurement errors, actual physical faults in the equipment, or unseen operating conditions.

To detect anomalies, the DSRF focuses on reconstructing the normal structural component explained by the normal operating structure while it simultaneously separates a sparse anomaly residual that cannot be explained by the learned normal model. To achieve this, the proposed DSRF jointly estimates the sparse coefficient vector α, which represents the normal structural representation, and the sparse abnormal residual e.

### 3.1. Construction of the Normal Observation Deviation Matrix

The sensor observed data matrix $Y_{S}\in R^{m\times S}$ is collected under normal operating conditions, where S denotes the number of normal training samples.

(15) $$Y_{S}=\left[y_{1},\;y_{2},\;y_{3},\ldots ,y_{S}\right]$$

The observation deviation in the i-th normal training sample is generated by calculating deviations from the reference observation vector and is then defined as

(16) $${\Delta y}_{i}=y_{i}-y_{ref}$$

where $y_{ref}$ denotes a reference observation vector and is defined from normal measurements. The normal observation deviation matrix $Y_{∆}\in R^{m\times S}$ is constructed as

(17) $$Y_{∆}=\left[{∆y}_{1},\;{∆y}_{2},\;{∆y}_{3},\ldots ,{∆y}_{S}\right]$$

This matrix is used to learn the effective sensing matrix directly in the observation space.

### 3.2. Data-Driven Effective Sensing Matrix Learning

The effective sensing matrix Φ is learned directly from the normal observation deviation matrix $Y_{∆}$. The learning problem of the effective sensing matrix is formulated by jointly minimizing the reconstruction error and the sparsity regularization term as follows:

(18) $$\underset{\Phi ,\Gamma }{\min}\{\frac{1}{2}\|Y_{∆}-\Phi \Gamma {\|}_{F}^{2}+\lambda_{\Phi }{\left\|\Gamma \right\|}_{1}\},\;\mathrm{Subject}\;\mathrm{to}\;\;{\left\|\phi_{k}\right\|}_{2}\leq 1,\;k=1,\ldots ,K$$

where $\Phi =\left[\phi_{1},\;\phi_{2},\;\phi_{3},\ldots ,\phi_{K}\right]\in R^{m\times K}$ denotes the learned effective sensing matrix, $\phi_{k}$ denotes its k-th column, and $\Gamma =\left[\gamma_{1},\;\gamma_{2},\;\gamma_{3},\ldots ,\gamma_{S}\right]\;\in R^{K\times S}$ is referred to as the sparse coefficient matrix where each column $\gamma_{i}$ corresponds to the i-th normal training sample. The parameter $\lambda_{\Phi }$ controls the strength of the sparsity regularization applied to the sparse coefficient matrix Γ.

The normalization constraint on each column of the learned effective sensing matrix Φ prevents the scale ambiguity between the effective sensing matrix and the sparse coefficients Γ. Without this constraint, the same observation deviation could be reconstructed by arbitrarily scaling Φ and inversely scaling Γ. This is why it leads in non-unique solutions. The learned effective sensing matrix captures recurring structural patterns of the normal observation deviations in the sensor observation space. Consequently, each normal observation deviation can be represented using only a limited number of columns of the effective sensing matrix Φ and is expressed as

(19) $${∆y}_{i}\approx \Phi \gamma_{i}.$$

This sparse representation provides an effective low-dimensional description of the normal observation deviation structure of the sensor data. The learned effective sensing matrix directly maps the sparse coefficient vector to the reconstructed normal observation deviation in the sensor observation space.

### 3.3. Joint Sparse Inverse Optimization

For a newly observed sensor vector y, the observation deviation from the reference vector is defined as $∆y={y-y}_{ref}$, where the reference observation vector $y_{ref}$ is calculated directly from the normal training measurements. The proposed DSRF jointly estimates the sparse coefficient vector α, which determines the sparse representation of the normal observation deviation, and the sparse anomaly residual e by solving the optimization problem:

(20) $$\underset{\alpha ,e\;}{\min}\lambda_{\alpha }{\left\|\alpha \right\|}_{1}+\lambda_{e}{\left\|e\right\|}_{1},\;subject\;to\;{\left\|\Delta y-\Phi \alpha -e\right\|}_{2}\leq \epsilon$$

The first regularization term promotes a sparse representation of the normal observation deviation using only a limited number of columns of the learned effective sensing matrix. The second regularization term promotes sparsity of the anomaly residual based on the assumption that abnormal behavior occurs locally in a limited number of sensors or measurement features.

The parameters $\lambda_{\alpha }$ and $\lambda_{e}$ control the trade-off between the sparsity representation of the normal observation deviation and the sparsity of the anomaly residual. The parameter ϵ is defined as the allowable reconstruction residual caused by modeling errors and unmodeled measurement uncertainties.

Note that the small variation term η in Equation (14) is not explicitly estimated and its magnitude is accommodated by the constraint in Equation (20). Equation (20) is solved using a standard primal-dual convex optimization algorithm, specifically the Chambolle–Pock primal–dual algorithm, which explicitly enforces the residual constraint through projection onto the $\;l_{2}$-ball defined by the parameter ϵ. The $l_{1}$-norm variables are updated using proximal soft-thresholding operators, and the primal and dual step sizes are selected to satisfy the standard convergence condition.

After solving the joint sparse inverse optimization problem, the observation deviation Δy can be decomposed into the reconstructed normal measurement component, the estimated sparse anomaly residual, and the remaining reconstruction error as follows:

(21) $$\Delta y=\Phi \hat{\alpha }+\hat{e}+r$$

where $\Phi \hat{\alpha }$ is the reconstructed normal measurement component, $\hat{e}$ is the estimated sparse anomaly residual and r is the remaining reconstruction residual satisfying ${\left\|r\right\|}_{2}\leq \epsilon$.

Therefore, the proposed DSRF identifies sparse abnormal components in the sensor observation space while it reconstructs the normal measurement component consistent with the learned operating structure.

### 3.4. Anomaly Residual-Based Anomaly Scoring and Auxiliary Reliability Indicator

The estimated anomaly residual $\hat{e}$ represents measurement components that cannot be adequately explained by the effective sensing matrix learned from normal sensor measurements. For the i-th measurement or feature, the feature-level anomaly score $s_{i}$ is defined as ${AS}_{i}^{f}=\left|{\hat{e}}_{i}\right|$. A larger value of ${AS}_{i}^{f}$ indicates that the corresponding measurement is less consistent with the normal operating model. Therefore, the larger the ${AS}_{i}^{f}$, the stronger the indication of an anomaly.

When multiple features correspond to one sensor, a sensor-level anomaly score can be defined as ${AS}_{j}^{s}={\left\|\hat{e}I_{j}\right\|}_{2}$, where $I_{j}$ denotes the set of feature indices related to the j-th sensor (i.e., $I_{j}=\left\{i\in \left\{1,2,\ldots ,m\right\}\right|$ the i-th measurement feature is extracted from sensor j}). The sensor-level anomaly score is then defined as

(22) $${AS}_{j}^{s}={\left\|\hat{e}I_{j}\right\|}_{2}=\sqrt{\sum_{i\in I_{j}}{\hat{e}}_{ii}^{2}}$$

In this regard, here, $\hat{e}I_{j}$ denotes the sub-vector of the estimated anomaly residual $\hat{e}$ containing only the features related to the j-th sensor.

For a sensor-level anomaly score ${AS}_{j}^{s}$, a normalized reliability score ${RS}_{j}$ of j-th sensor can be defined as

(23) $${RS}_{j}=\frac{1}{1+\rho {AS}_{j}^{s}},$$

where ρ denotes a sensitivity parameter that controls the sensitivity of the reliability score to the anomaly score and is greater than zero (i.e., ρ>0). This reliability score for the j-th sensor lies in the range $0<{RS}_{j}\leq 1$. Therefore, as the value of ${AS}_{j}^{s}$ increases, the reliability score decreases and approaches zero. A larger reliability score therefore indicates greater consistency with the learned normal operating structure while a smaller value indicates a larger unexplained abnormal component. Therefore, ${RS}_{j}$ should be interpreted as a normalized indicator of consistency with the learned normal measurement structure rather than as a directly validated physical reliability measure.

A sensor-level abnormal condition for the j-th sensor can be identified when ${AS}_{j}^{s}>\tau_{s}$, equivalently when ${RS}_{j}<\tau_{R}$, where $\tau_{s}$ and $\tau_{R}$ are thresholds determined exclusively from validation data collected under normal operating conditions. In the present study, however, the reliability indicator itself is not independently validated against physical sensor health labels, degradation levels, or failure probabilities.

In addition to the sparse anomaly residual, the remaining reconstruction residual may be used as a supplementary system-level anomaly indicator: $S_{e}={\left\|\Delta y-\Phi \hat{\alpha }-\hat{e}\right\|}_{2}$. The remaining reconstruction residual represents components that are not sufficiently represented by either the learned normal structure or the sparse anomaly residual. In the present study, the primary anomaly decision is based on the anomaly score derived from the estimated anomaly residual, while the sensor-level and reliability indicators are introduced only as auxiliary quantities for supplementary sensor-level interpretation. Independent validation of this reliability indicator is beyond the scope of the present study and will be investigated in future work.

The sensor-level anomaly score derived from the estimated anomaly residual and the reconstruction residual have different interpretations. The sensor-level anomaly score measures localized components separated from the normal model while the reconstruction residual measures the remaining component that is not sufficiently represented by either the normal structure or the sparse anomaly residual. In this regard, the system-level anomaly indicator is important for detecting system change caused by unseen operating conditions and simultaneous changes in multiple sensors.

### 3.5. Discussion and Distinctive Features of Proposed Framework

Existing dictionary learning-based anomaly detection methods generally learn sparse representations directly from sensor measurements and identify abnormal samples based on reconstruction errors [20,22]. Although these methods can effectively capture normal observation patterns, they commonly rely only on reconstruction error and do not explicitly separate a sparse abnormal component from the normal measurement representation.

First, the proposed DSRF differs from such methods by formulating equipment condition monitoring as a data-driven sparse linear inverse problem in the sensor observation space. Specifically, the theoretical formulation interprets the effective sensing matrix as Φ=AD, where A represents the physical sensing process and D represents the structural patterns of the effective state. However, it is noted that, in the practical implementation, the combined effect of A and D is constructed and refined from normal sensor measurements because the measurement matrix A and the effective state variables are not separately observable. The learned effective sensing matrix, therefore, provides a data-driven representation of the combined effects of the normal structural patterns and the sensor observation process. Furthermore, in the practical implementation, the sparse coefficients should be interpreted as coefficient vectors that reconstruct the normal measurement structure through the learned effective sensing matrix.

Second, the proposed DSRF explicitly introduces the sparse anomaly residual e and jointly estimates that e with the coefficient vector α. Existing reconstruction-based methods commonly use the total reconstruction error as an anomaly score. In contrast, the DSRF separates the observation deviation into a normal measurement component Φα, a sparse anomaly residual e, and a small uncertainty term η. This decomposition provides separate estimates of the reconstructed normal measurement component and the localized sparse anomaly residual e.

A large sparse anomaly residual does not necessarily indicate only a sensor fault. It may also be caused by an actual physical abnormality of the equipment, an unseen operating condition, and an unexpected disturbance. Therefore, the estimated sparse anomaly residual should mainly be interpreted as an indicator of inconsistency with the learned normal observation structure represented by the effective sensing matrix. Note that additional information, such as temporal persistence, relationships among sensors, redundant measurements, or physical constraints, may be required to distinguish sensor faults from actual equipment anomalies.

The key novelty of the proposed framework is therefore not merely the use of sparse representation or dictionary learning techniques. Rather, it lies in the reformulation of anomaly detection as a data-driven sparse linear inverse problem that constructs and refines an effective sensing matrix from normal sensor measurements and jointly estimates the normal measurement component and sparse anomaly residual.

## 4. Experimental Evaluation

### 4.1. Experimental Dataset

The proposed DSRF is evaluated using current and vibration measurements collected from a robotic welding system, which include both normal data and abnormal data caused by irregularly occurring anomalies [34]. In actual industrial settings, failures occur much less frequently than normal operation. It makes it difficult to collect a sufficient amount of abnormal sensor data. In addition, intentionally operating equipment under faulty conditions to collect data is subject to practical constraints in terms of safety and cost. Accurately identifying and labeling the failure types of collected abnormal signals also requires specialized knowledge and considerable resources. Consequently, real-world industrial datasets often exhibit a highly imbalanced distribution, with normal samples accounting for the majority of the data and abnormal samples representing only a small proportion. The dataset used in this study reflects this practical imbalance because the data were collected from actual industrial operations rather than from artificially induced fault conditions.

The current dataset consists of 8202 normal samples and 32 abnormal samples. Each current sample contained 1024 frequency-domain features. The vibration dataset consists of 1774 normal samples and 16 abnormal samples, with each sample containing 512 frequency-domain features. The datasets are summarized in Table 1.

The normal data are divided into training, validation, and independent test subsets at an approximate ratio of 60:20:20. Specifically, the 8202 normal current samples are divided into 4921 training samples, 1640 validation samples, and 1641 independent test samples, while the 1774 normal vibration samples are divided into 1064 training samples, 355 validation samples, and 355 independent test samples.

Only normal measurements are used for model construction and threshold determination. The training subset is used to construct and refine the effective sensing matrix while the validation subset is used exclusively to determine the anomaly threshold $\tau_{p}$. The independent test subset is used for final evaluation of false positive-based performance. All 32 abnormal current samples and 16 abnormal vibration samples are reserved exclusively for final performance evaluation. Therefore, for final performance evaluation, the independent normal test samples and the labeled abnormal samples are evaluated together to calculate the confusion matrix and performance metrics, including the false positive rate, false negative rate, detection rate, and accuracy.

### 4.2. Data Processing

The remaining columns, except for the first column containing the measurement time of each sample, are treated as frequency-domain measurement features.

First, since the spectral amplitudes exhibit highly skewed distributions and substantially different magnitudes across frequency components, the non-negative feature values are transformed using $z_{ij}=log({1+y}_{ij})$, where $y_{ij}$ denotes the j-th feature of the i-th measurement sample. Second, after the logarithmic transformation, percentile-based scaling is applied using the 10th and 90th percentiles of the normal training data. The scaled j-th feature value of the i-th measurement sample is defined as

(24) $${\bar{z}}_{ij}=\frac{z_{ij}-c_{j}}{q_{90,j}-q_{10j}}$$

where $c_{j}$ denotes the center value used for the j-th feature and $q_{90,j}$ and $q_{10j}$ denote the 10th and 90th percentiles, respectively, of the j-th feature in the normal training set. The same scaling parameters $q_{90,j}$ and $q_{10j}$ estimated exclusively from the normal training set are then applied without modification to the normal validation data and abnormal test data.

Therefore, the preprocessed observation vector ${\bar{z}}_{i}$ for the i-th measurement sample is constructed as ${\bar{z}}_{i}={\left[{\bar{z}}_{i1},{\bar{z}}_{i2},\ldots ,\;{\bar{z}}_{im}\right]}^{T}$. It denotes the preprocessed version of the i-th sensor observed vector $y_{i}$, obtained after logarithmic transformation and percentile-based scaling.

Third, the reference observation vector ${\bar{z}}_{ref}$ is calculated as the mean of the preprocessed normal training observation vectors,

(25) $${\bar{z}}_{ref}=\frac{1}{S}\sum_{i=1}^{S}{\bar{z}}_{i},$$

where S denotes the number of normal training samples. The observation deviation $\Delta {\bar{z}}_{i}$ of the i-th normal training sample is defined as

(26) $$\Delta {\bar{z}}_{i}={\bar{z}}_{i}-{\bar{z}}_{ref}$$

This procedure allows the model to represent deviations from the normal measurement structure rather than the absolute magnitude of each frequency-domain feature.

### 4.3. Constructing the Effective Sensing Matrix

As discussed in Section 3, the physical measurement matrix A and the state space dictionary matrix D are not separately identifiable from the available sensor datasets. Therefore, their combined effect is represented by an effective sensing matrix in the observation space.

In this experiment, PCA is first applied to the normal observation deviation matrix to obtain an initial compact representation of the dominant normal structure. As defined in Section 3.2, the effective sensing matrix is defined as $\Phi =\left[\phi_{1},\;\phi_{2},\;\phi_{3},\ldots \phi_{K}\right]$, where each column is a basis vector learned from the normal training data. For the current dataset, 64 principal components are retained and result in an initial matrix $\Phi_{c}^{0}\in R^{1024\times 64}$. For the vibration dataset, 48 principal components are retained and result in $\Phi_{v}^{0}\in R^{512\times 48}$. The retained components explain approximately 87.44% of the variance in the normal current observation deviations and 68.89% of the variance in the normal vibration observation deviations. These PCA bases are used only as initial representations and are not regarded as the final effective sensing matrices. Therefore, PCA is first applied to the normal measurement deviations to obtain an initial low-dimensional representation $\Phi^{0}$.

While it starts from PCA initialization, the effective sensing matrix is refined according to the sparse representation objective defined in Equation (18). The sparse coefficient matrix Γ and the effective sensing matrix Φ are alternately updated to reconstruct the normal observation deviations using a limited number of active coefficients. Consequently, the final effective sensing matrix is determined through the sparse refinement defined in Equation (18). The refined effective sensing matrix is subsequently used in the joint sparse inverse optimization for anomaly separation.

### 4.4. Joint Sparse Reconstruction and Anomaly Separation

For each observation deviation Δy, the proposed DSRF jointly estimates the sparse normal coefficient vector α and the sparse anomaly residual e based on the model in Equation (14). The sparse components, α and e, are estimated by solving Equation (20) using the refined effective sensing matrix, subject to a constraint on the remaining reconstruction residual.

In this experiment, the parameter ϵ, which specifies the allowable magnitude of the remaining reconstruction residual, is determined from the reconstruction residual distribution of the normal training data after refinement of the effective sensing matrix. Specifically, the 95th percentile of the reconstruction residual norms obtained from the normal training data is used to define the allowable residual level. This data-driven selection relates the residual constraint to the variability observed under normal operating conditions rather than assigning ϵ arbitrarily.

In the numerical implementation, Equation (20) is solved using a primal–dual convex optimization algorithm with the refined effective sensing matrix. The reconstruction residual constraint defined by ϵ is explicitly incorporated into the optimization. The sparse coefficient vector α and the anomaly residual e are jointly estimated using the primal–dual updates. The iterative optimization is terminated when the relative change between successive iterates falls below a predefined tolerance or when the maximum number of iterations is reached.

After solving Equation (20), each observation deviation is decomposed according to Equation (21), and the resulting estimates $\hat{\alpha }$ and $\hat{e}$ represent the sparse normal coefficient vector and the anomaly residual, respectively. Therefore, the anomaly residual is explicitly estimated through the joint sparse inverse optimization rather than being defined simply as the residual remaining after PCA-based reconstruction.

In Equation (20), the parameter $\lambda_{\alpha }$ controls the sparsity of the coefficient vector α which represents the normal measurement structure through the refined effective sensing matrix. A smaller $\lambda_{\alpha }$ allows more coefficient components to remain active. If it is excessively small, abnormal patterns may be partially represented by the reconstructed normal component, resulting in a reduced anomaly residual. A larger $\lambda_{\alpha }$ promotes a sparser coefficient vector by retraining fewer active component. However, an excessively large $\lambda_{\alpha }$ may suppress the normal coefficient representation too strongly. It results in larger reconstruction residuals even for normal samples. The parameter $\lambda_{e}$ controls the sparsity of the anomaly residual e. A smaller $\lambda_{e}$ allows relatively small unexplained components to be included in the anomaly residual, while a larger $\lambda_{e}$ promotes a sparser anomaly residual by retaining only components with relatively large magnitudes. Therefore, $\lambda_{\alpha }$ and $\lambda_{e}$ jointly control the allocation of the observation deviation between the reconstructed normal measurement component and the anomaly residual.

To evaluate the sensitivity of the joint sparse inverse optimization to these regularization parameters, the values of both $\lambda_{\alpha }$ and $\lambda_{e}$ are varied over predefined ranges. In the sensitivity analysis, multiple combinations of both parameters are evaluated. Specifically, $\lambda_{\alpha }\in \left\{0.01,\;0.05,\;0.2\right\}$ and $\lambda_{e}\in \left\{0.25,\;1.0,\;2.5,\;8.0\right\}$ are evaluated. For each parameter combination, the anomaly threshold $\tau_{p}$ is recalculated as the 99.5th percentile of the anomaly scores obtained from the normal validation data. The labeled abnormal samples are used only to evaluate the resulting detection performance for the sensitivity analysis and are not used as a criterion for selecting the regularization parameters. The normal validation subset is used only for threshold determination while the independent normal test subset is used to calculate the false positive rate and other false positive-based performance measures. The labeled abnormal samples are reserved for the evaluation of true positives and false negatives.

For the current dataset, all tested parameter combinations result in no false negatives, while the number of false positives varies only slightly from two to five. The corresponding accuracy remains between 0.99701 and 0.99880. Although the anomaly threshold varies according to the values of $\lambda_{\alpha }$ and $\lambda_{e}$, the overall classification performance remains consistently high over the tested parameter combinations. For example, when $\lambda_{e}$ is 1.0, changing $\lambda_{\alpha }$ from 0.01 to 0.20 changes the anomaly threshold from 0.185688 to 0.187549, while the numbers of false positives only change from three to four and no false negatives are observed.

These results indicate that anomaly detection performance is relatively insensitive to the tested values of $\lambda_{\alpha }$ and $\lambda_{e}$ when the refined effective sensing matrix is used. In particular, no false negatives are observed for any of the tested parameter combinations. For the current dataset, only small variations in the number of false positives are observed, while the vibration classification results remain unchanged over the entire tested range.

The representative parameter settings are determined independently of the labeled abnormal-sample results and are subsequently fixed for final performance evaluation. The values of $\lambda_{\alpha },$ which is set to 0.05, and $\lambda_{e},$ which is set to 1.0, are used for the current measurements, while $\lambda_{\alpha }$ given as 0.05 and $\lambda_{e}$ given as 2.5 are used for the vibration measurements.

The value of $\lambda_{\Phi }$ is determined empirically using only the normal training data by considering the trade-off between reconstruction fidelity and coefficient sparsity during refinement of the effective sensing matrix. A value that provides sufficient coefficient sparsity without markedly increasing the reconstruction residual of the normal training data is selected. Accordingly, $\lambda_{\Phi }$, which is set to 0.05, is used for the current data while $\lambda_{\Phi }$, which is set to 0.5, is used for the vibration data.

The allowable reconstruction residual level ϵ is determined from the 95th percentile of the reconstruction residual norms obtained from the normal training data. Under the revised 60:20:20 data partition, ϵ, which is set to 13.893707, is used for the current dataset and ϵ, which is set to 8.978036, is used for the vibration dataset. The experimental parameter settings are summarized in Table 2.

### 4.5. Anomaly Score and Threshold Selection

For each measurement sample, the sample-level anomaly score is calculated from the estimated anomaly residual as

(27) $${AS}^{sp}=\frac{{\left\|\hat{e}\right\|}_{2}}{\sqrt{m}},$$

where m is the number of measurement features. Division by $\sqrt{m}$ is used to reduce the direct dependence of the anomaly score on the number of features. A larger value of ${AS}^{sp}$ indicates that a larger portion of the observation deviation is separated as an anomaly residual rather than being represented by the normal measurement structure through the refined effective sensing matrix.

The anomaly threshold $\tau_{p}$ is determined using only the anomaly scores obtained from the normal validation data. Multiple threshold percentiles are evaluated, with p∈{95, 97.5, 99.5, 99.9}. For each percentile p, the anomaly threshold $\tau_{p}$ is calculated as $\tau_{p}=Q_{p}\left({AS}_{1}^{sp},{AS}_{2}^{sp},\ldots ,{AS}_{N_{val}}^{sp}\right),$ where $N_{val}$ is given as {$N_{c\_val},\;N_{v\_val}$} = {1640, 355} and $Q_{p}(\cdot )$ denotes the p-th percentiles of the normal validation anomaly scores. $N_{c\_val}$ denotes the number of current validation samples. The number of vibration validation samples is referred to as $N_{v\_val}$.

A measurement sample is classified as an anomaly when

(28) $${AS}^{sp}>\tau_{p}.$$

After the threshold is determined from the normal validation subset, it is fixed and subsequently applied to the independent normal test samples and the labeled abnormal samples for final performance evaluation.

### 4.6. Experimental Results

Using the DSRF, the performance of anomaly detection for robot welding systems in smart manufacturing environments is evaluated in terms of TP (true positive), TN (true negative), FP (false positive), and FN (false negative) rates.

**Figure 1:**
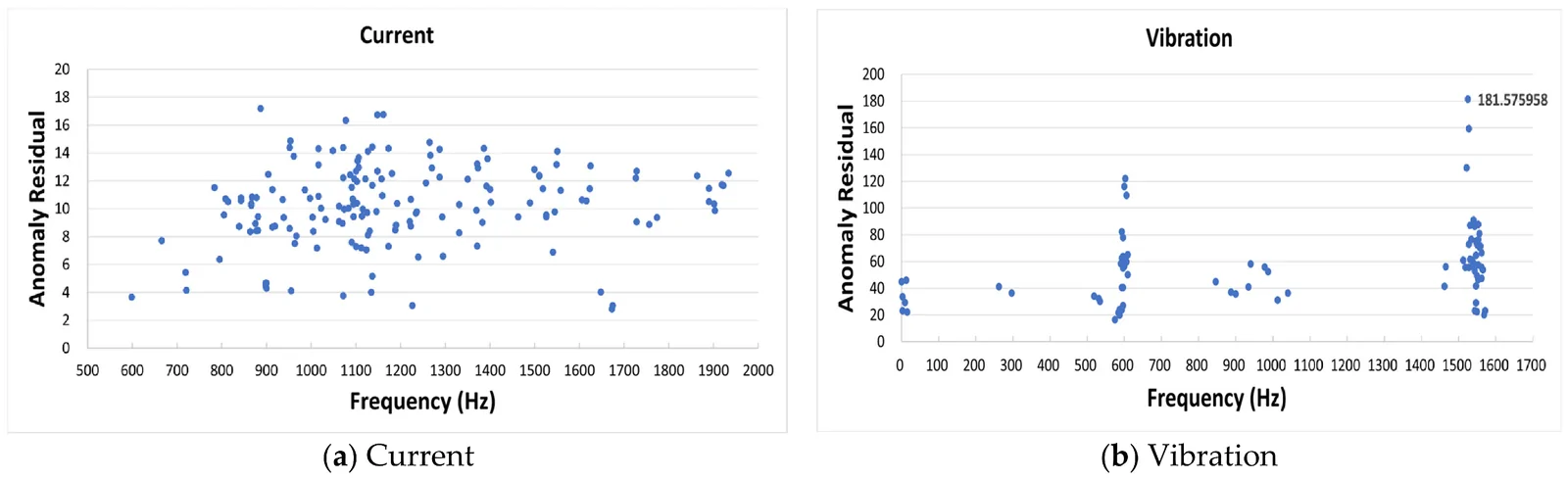
Distributions of the five largest absolute anomaly residuals across frequency features for abnormal samples: (**a**) 160 anomaly residual points from 32 abnormal current samples; (**b**) 80 anomaly residual points from 16 abnormal vibration samples.

Figure 1 shows the distributions of the five frequency features with the largest absolute anomaly residuals for each abnormal sample. Selecting the top five features for each abnormal sample yields 160 anomaly residual points for the 32 abnormal current samples and 80 points for the 16 abnormal vibration samples.

In the current samples, the dominant frequencies of the anomaly residuals are widely distributed between approximately 600 and 1930 Hz as shown in Figure 1a, and the dominant frequency varies among abnormal samples. This indicates that the abnormal measurements are not characterized by a single fixed frequency component. In the current measurements, relatively large anomaly residuals appear across a substantial number of frequency features. This means that the frequency domain characteristics related to the detected abnormalities vary from sample to sample.

The vibration anomalies exhibit large anomaly residuals in both low- and high-frequency regions. The detected frequency features and their anomaly residual can therefore provide supplementary information for interpreting the abnormal measurement patterns. As shown in Figure 1b, in the vibration samples, large anomaly residuals are observed over a wide frequency range, with the low frequency range below approximately 500 Hz, the middle frequency range from approximately 500 to 1000 Hz, and the high frequency range from 1000 Hz to 1570 Hz. These results indicate that the vibration anomalies are characterized by anomalies distributed across multiple frequencies rather than a single dominant frequency. The largest anomaly residual is observed in the high-frequency region, approximately 1525 Hz, with a maximum value of approximately 181.58. The presence of the largest anomaly residual in the high-frequency region indicates that prominent abnormal deviations are observed in the high-frequency components for some detected abnormalities. At the same time, the sparse anomaly residual provides useful feature-level information for identifying frequency regions associated with abnormal measurement patterns.

When the anomaly threshold is set to the 99.5th percentile of the normal validation scores, the threshold for the current dataset is 0.185676. Among the 1641 independent normal test samples, four samples are incorrectly classified as anomalies that correspond to a false positive rate of approximately 0.24%. All 32 abnormal current samples are correctly detected. This results in a detection rate of 100% and a false negative rate of 0%. For the vibration dataset, the 99.5th-percentile anomaly threshold $\tau_{p}$ determined from the normal validation scores is 0.368756. A total of 6 of the 355 independent normal test samples are incorrectly classified as anomalies, corresponding to a false positive rate of approximately 1.69%. All 16 abnormal vibration samples are correctly detected. This results in a detection rate of 100% and a false negative rate of 0%. These results are summarized in Table 3. The detection rate is defined as the percentage of actual abnormal samples correctly identified as anomalies.

**Figure 2:**
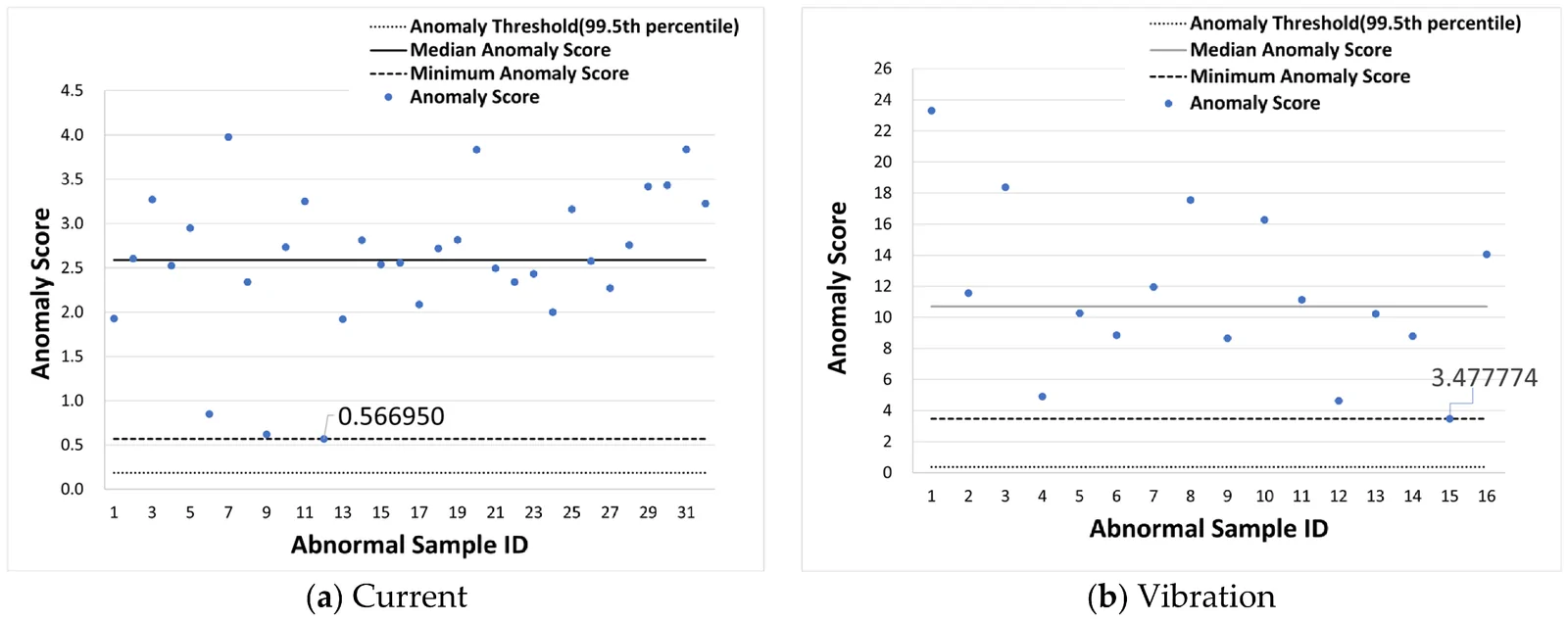
The distribution of sample-level anomaly scores with the anomaly threshold determined from the 99.5th percentile of the normal validation data: (**a**) current; (**b**) vibration.

Figure 2 shows the distributions of the sample-level anomaly scores with the anomaly thresholds determined from the 99.5th percentile of the normal validation scores, as summarized in Table 3. Under normal operating conditions, most of the observation deviation can be represented by the reconstructed normal measurement component $\Phi \hat{\alpha }$ through the refined effective sensing matrix, while the estimated anomaly residual $\hat{e}$ remains relatively small. In contrast, the abnormal measurements contain frequency-domain patterns that cannot be sufficiently represented by the learned normal measurement structure. Through the joint sparse inverse optimization, these components are separated into the estimated anomaly residual. This results in larger anomaly scores. Consequently, anomaly scores are significantly increased since these patterns are separated into the sparse anomaly residual. Therefore, the anomaly score of the proposed DSRF provides clear separation between the evaluated abnormal samples and the decision threshold derived from the normal validation data.

As shown in Figure 2a, for the current measurements, the minimum and median anomaly scores of the 32 abnormal samples are 0.566950 and 2.589786, respectively, both of which are substantially greater than the designated anomaly threshold $\tau_{p}$ of 0.185676 reported in Table 3. The minimum anomaly score corresponds to the abnormal sample with the lowest anomaly score among the evaluated abnormal samples. These results indicate that all evaluated abnormal samples remain above the selected anomaly threshold.

Similarly, as shown in Figure 2b, the minimum and median anomaly scores of the 16 abnormal vibration samples are 3.477774 and 10.702208, respectively, while the corresponding threshold $\tau_{p}$ is 0.368756, as summarized in Table 3. All evaluated abnormal vibration samples are therefore located above the selected threshold. The relatively large anomaly scores reflect the larger magnitudes of the estimated anomaly residuals for these samples.

Table 4 compares the anomaly detection results for the current and vibration datasets using different percentile thresholds determined from the normal validation score distributions and evaluated on the independent test data. For both the current and vibration datasets, increasing the anomaly threshold percentile consistently reduces the number of false positives. There are no false negatives at any of the evaluated anomaly thresholds. The 99.9th percentile threshold produces the lowest false positive rate with no false positives for the current data and four false positive for the vibration dataset while it maintains a detection rate of 100% for both datasets. These results indicate that the sample-level anomaly score provides stable discrimination between the independent normal test samples and the evaluated abnormal samples over the tested threshold percentiles. Furthermore, increasing the anomaly threshold percentile substantially reduces the false positive rate without any false negatives. This indicates that the anomaly scores of the evaluated abnormal samples remain well separated from those of the independent normal test samples across all evaluated anomaly threshold percentiles.

The experimental results presented in Figure 1 and Figure 2 and Table 3 and Table 4 support the feasibility of constructing an initial effective sensing matrix from normal sensor measurements and subsequently refining it according to the sparse representation objective. This provides a practical implementation of the proposed data-driven sparse inverse framework when the physical measurement matrix and effective state variables are not separately observable. The estimated anomaly residual also provides feature-level information by identifying the frequency features related to the largest abnormal deviation. Therefore, the proposed DSRF not only determines whether a measurement is anomalous, but also identifies which frequency features exhibit the largest anomaly residuals and quantifies their corresponding magnitudes.

The evaluated abnormal samples remain clearly separated from the independent normal test samples, and no false negatives are observed for any of the tested threshold percentiles. However, it is noted that the number of labeled abnormal samples is limited to 32 current samples and 16 vibration samples.

To evaluate robustness to changes in the normal-data partition, the proposed DSRF is repeatedly evaluated using multiple random seeds and three train–validation–test ratios, namely 60:20:20, 65:15:20, and 70:10:20. As shown in Table 5, the detection rate remains 100% across all evaluated train–validation–test ratios and random splits for both the current and vibration datasets. Although the false positive rate varies depending on the data partition, no false negatives are observed in any of the evaluated settings. These results indicate that the abnormal-sample detection outcome remains consistent under moderate changes in both the proportion and composition of the normal training data.

### 4.7. Comparison of Experimental Results

In this paper, we use PCA and autoencoder reconstruction as baseline methods to assess the effectiveness of the proposed DSRF against representative linear and nonlinear reconstruction-based anomaly detection approaches. PCA provides a conventional linear reconstruction baseline while the autoencoder represents a nonlinear deep learning approach. All three methods are evaluated under the same preprocessing, data partition, and threshold setting conditions to provide a consistent basis for comparison. A total of five experiments are conducted using different random splits of the normal measurements.

In each experiment, the normal measurements are divided into training, validation, and independent test subsets at an approximate ratio of 60:20:20. For the current dataset, 4921 normal samples are used for training, 1640 for validation, and 1641 for independent testing. For the vibration dataset, 1064 normal samples are used for training, 355 for validation, and 355 for independent testing. The sample counts are maintained across the five experiments, as summarized in Table 6. The training subset is used for model construction, the validation subset is used exclusively to determine the anomaly threshold, and the independent normal test subset is used for final false positive-based performance evaluation. The 32 abnormal current samples and 16 abnormal vibration samples are not used for model construction, parameter estimation, or threshold determination. They are used only for final performance evaluation.

PCA reconstruction, an autoencoder reconstruction model, and the proposed DSRF are evaluated under the same preprocessing and data partitioning conditions. For each method, the anomaly threshold is determined as the 99.5th percentile of the corresponding normal validation anomaly score distribution. For the DSRF, as mentioned above, PCA is used to construct the initial effective sensing matrix, which is subsequently refined according to Equation (18). The refined effective sensing matrix is then used in the joint sparse inverse optimization in Equation (20) to estimate the normal coefficient vector and the anomaly residual.

Under the 60:20:20 experimental setting, PCA reconstruction, the autoencoder, and the DSRF all detect the evaluated abnormal samples without false negatives, as shown in Table 7.

For the current measurements, all three methods correctly detect all 32 abnormal samples without false negatives. Among 1641 independent normal test samples, PCA correctly classifies 1638 samples as normal and incorrectly classifies three samples as abnormal. This results in an accuracy of 99.82%. The autoencoder and the DSRF each correctly classify 1637 normal samples and incorrectly classify 4 samples as abnormal, resulting in an accuracy of 99.76%.

For the vibration measurements, all three methods yield the same confusion matrix results. Among the 355 independent normal test samples, 349 samples are correctly classified as normal and 6 are incorrectly classified as abnormal. All 16 abnormal samples are correctly detected without false negatives, resulting in an accuracy of 98.38% for all three methods.

These results show that PCA reconstruction, the autoencoder, and the proposed DSRF achieve comparable classification performance at the selected threshold percentile for the evaluated datasets. All three methods detect all evaluated abnormal samples while only small differences in the number of false positives are observed for the current dataset. In this regard, the distinction among the methods is not primarily reflected in the detection rate, but rather in other characteristics such as the separation of anomaly scores and the explicit estimation of the anomaly residual provided by DSRF.

To examine the anomaly score separation characteristics and their stability across repeated experiments for the three methods, the analysis results are summarized in Table 8. To quantify the separation between the abnormal sample with the lowest anomaly score and the decision threshold, we introduce the minimum anomaly-to-threshold ratio (MATR), defined as

(29) $$MATR=\frac{\underset{i\in A}{\min}{AS}_{i}^{sp}}{\tau_{p}}$$

where A denotes the set of abnormal samples. MATR represents the ratio between the lowest anomaly score among the evaluated abnormal samples and the corresponding anomaly threshold $\tau_{p}$. Therefore, MATR quantifies the threshold-normalized separation between the abnormal sample with the lowest anomaly score and the corresponding anomaly threshold. An MATR greater than 1 indicates that all evaluated abnormal samples have anomaly scores above the threshold. A larger MATR indicates a greater separation between the lowest abnormal score and the threshold.

As shown in Table 8, all three methods achieve a very high AUROC and average precision values over the five repeated experiments, indicating similar ranking performance for the evaluated datasets. The proposed DSRF yields the largest mean MATR for both the current and vibration measurements. For the current dataset, the mean MATR values are 1.631, 1.785, and 3.159 for PCA, the autoencoder, and the DSRF, respectively.

For the vibration measurements, the mean MATR of the DSRF is also the largest at 4.461, compared with 3.211 for PCA and 3.325 for the autoencoder. However, the DSRF also exhibits the largest standard deviation of 3.103, compared with 1.642 for PCA and 1.741 for the autoencoder. This result indicates a larger mean separation from the decision threshold for the DSRF, though the larger standard deviation indicates greater variability in this separation across the different data splits.

These results indicate that although the three methods achieve similarly high classification and ranking performance on the evaluated datasets, the DSRF provides a larger threshold-normalized separation between the evaluated abnormal samples and the decision threshold.

### 4.8. Comparison of Residual Characteristics Between PCA and DSRF

Table 9 compares the Hoyer sparsity and top-k feature concentration for the PCA reconstruction residual, DSRF anomaly residual, and DSRF remaining reconstruction residual in the current and vibration datasets. Hoyer sparsity is a widely used sparsity measure that quantifies the degree of sparsity of a vector on a scale from 0 to 1, where a value closer to 1 indicates that the vector energy is concentrated in a smaller number of components. Top-k concentration is defined as the ratio of the sum of the k largest absolute residual magnitudes to the total absolute residual magnitude.

In PCA, all components not explained by the learned normal subspace are included in a single reconstruction residual. Consequently, this residual may contain not only local components associated with actual abnormal measurements but also factors such as imperfect representations of normal structures, modeling errors, and minor measurement fluctuations. In contrast, the DSRF explicitly decomposes the observed deviation into a component represented by the learned normal structure, a sparse anomaly residual, and a remaining reconstruction residual. Sparsity regularization encourages relatively concentrated unexplained components to be represented in the anomaly residual, while the remaining discrepancy is retained in the reconstruction residual.

The experimental results support the difference in residual characteristics between PCA reconstruction and the proposed DSRF. For the current dataset, Hoyer sparsity increases from 0.240 for the PCA reconstruction residual to 0.362 for the DSRF anomaly residual, while for the vibration dataset it increases from 0.281 to 0.397. Similarly, the top-10 concentration increases from 4.10% to 5.86% for the current dataset and from 9.80% to 13.53% for the vibration dataset. In contrast, the Hoyer sparsity of the DSRF remaining reconstruction residual is substantially lower, with values of 0.042 for the current dataset and 0.025 for the vibration dataset.

Therefore, compared with the PCA reconstruction residual, the DSRF anomaly residual exhibits higher Hoyer sparsity and greater top-k feature concentration for both current and vibration measurements. These results indicate that the unexplained components represented by the DSRF anomaly residual are concentrated in a relatively smaller number of frequency features while the remaining reconstruction residual is more diffusely distributed across the frequency features. The substantially lower Hoyer sparsity and top-k concentration of the remaining reconstruction residual further indicate that the residual mismatch left after anomaly separation is broadly distributed rather than concentrated in a few dominant features.

These characteristics are consistent with the intended decomposition of the DSRF. Unlike PCA reconstruction, in which all components not represented by the learned normal subspace are included in a single reconstruction residual, the DSRF explicitly decomposes the observed deviation into a reconstructed normal component, a sparse anomaly residual, and a remaining reconstruction residual. Through the joint sparse inverse optimization, relatively concentrated unexplained components are encouraged to be represented in the anomaly residual while the remaining discrepancy is retained in the reconstruction residual. Therefore, the DSRF does not simply treat all reconstruction mismatch as an anomaly. Small modeling errors, normal measurement variations, and other diffuse discrepancies can remain in the reconstruction residual while localized and relatively prominent deviations can be represented in the estimated anomaly residual.

Accordingly, the advantage of the DSRF over conventional PCA reconstruction is not necessarily reflected in higher classification accuracy because the three methods exhibit similarly high detection performance on the evaluated datasets. Rather, the DSRF provides a more explicit decomposition of the observation deviation. By calculating the anomaly score from the estimated anomaly residual rather than directly from the entire reconstruction mismatch, the influence of diffuse reconstruction mismatch on the anomaly score can potentially be reduced. In addition, because the anomaly residual is estimated under a sparsity constraint, prominent frequency-domain deviations can appear in a more concentrated form. This provides a more direct basis for feature-level interpretation by indicating which measurement features exhibit the largest deviations from the learned normal structure.

## 5. Conclusions and Future Work

This study proposed a data-driven sparse reconstruction framework (DSRF) for anomaly detection of sensor measurements. The proposed framework models an observation deviation as a combination of a component represented by the learned normal structure, a sparse anomaly residual, and a remaining reconstruction residual. The effective sensing matrix is initialized from normal sensor measurements using PCA and subsequently refined according to the sparse representation objective. The refined matrix is then used in the joint sparse inverse optimization to estimate the normal coefficient vector and anomaly residual without requiring separate identification of the physical measurement matrix or an explicit statistical noise model.

The proposed method is evaluated using current and vibration measurements from a robotic welding system. Only normal data are used for model construction and threshold determination while the labeled abnormal samples are reserved exclusively for final performance evaluation. PCA reconstruction and an autoencoder are also evaluated under the same preprocessing, data partitioning, and threshold setting conditions. All three methods achieve high classification and ranking performance on the evaluated datasets. In particular, the DSRF provides a larger threshold-normalized separation, as measured by the MATR, and explicitly estimates a sparse anomaly residual.

The residual characteristic analysis further shows that the DSRF anomaly residual exhibits higher Hoyer sparsity and greater top-k feature concentration than the PCA reconstruction residual, while the remaining reconstruction residual is more diffusely distributed. These results are consistent with the intended DSRF decomposition, in which relatively concentrated unexplained components are separated into the anomaly residual rather than all reconstruction mismatch being treated directly as anomalous.

Additional experiments using different normal-data partitions and random splits show that the evaluated abnormal samples are consistently detected without false negatives, although the false positive rate and threshold-normalized separation varies across splits. These results indicate that the detection outcome is relatively robust to moderate changes in the proportion and composition of the normal training data.

The present results demonstrate the feasibility of the proposed DSRF on the evaluated robotic welding dataset. However, the number of labeled abnormal samples is limited, and the current experiments do not establish generalization to independent machines, different operating conditions, near-normal or incipient anomalies, different fault severity levels, or previously unseen fault scenarios. Therefore, the current results do not establish generalization to independent machines, different operating conditions, near-normal or incipient anomalies, different fault-severity levels, or previously unseen fault scenarios. Further validation using more diverse industrial datasets and long-term operational data is required to assess broader generalization capability.

Future work should focus on extending the proposed DSRF to larger-scale sensor datasets collected under more diverse operating conditions. The current results demonstrate that the DSRF can effectively separate measurement deviations into a component explained by the learned normal structure and a sparse anomaly residual, while the residual provides feature-level information on abnormal measurement patterns. However, the present dataset does not include explicitly labeled fault severity levels or dedicated near-normal and incipient fault samples. Therefore, further validation using faults with different types and severity levels, particularly subtle abnormalities close to normal operating conditions, is required to evaluate the robustness and generalization capability of the framework. Furthermore, incorporating temporal characteristics and multi-sensor correlations into the sparse reconstruction framework will be explored to improve the detection capability for complex and evolving abnormal conditions.

## Figures and Tables

**Table 1:** Summary of current and vibration measurement datasets used for evaluation.

Signal Type	Status	Number of Samples
Current	Normal	8202
(1024 features)	Abnormal	32
Vibration	Normal	1774
(512 features)	Abnormal	16

**Table 2:** Summary of parameters configurated in experiment.

Parameter	Current	Vibration
Number of PCA initialization components	64	48
$\lambda_{\Phi }$	0.05	0.5
$\lambda_{\alpha }$	0.05	0.05
$\lambda_{e}$	1.0	2.5
ϵ	13.893707	8.978036

**Table 3:** Anomaly detection performance using thresholds determined from the normal validation data and evaluated on the independent normal test data and abnormal samples (99.5th percentile).

Type	$\tau_{p}$	TN	FP	FN	TP	Accuracy	Detection Rate
Current	0.185676	1637	4	0	32	99.76%	100%
Vibration	0.368756	349	6	0	16	98.38%	100%

**Table 4:** The effect of the anomaly threshold percentile on anomaly detection performance for the current and vibration datasets using independent normal test data and abnormal samples.

Type	Percentile	$\tau_{p}$	FP	FN	FPR ^1^	FNR ^2^	Accuracy
Current	95.0%	0.092655	75	0	4.57%	0%	95.56%
	97.5%	0.120491	35	0	2.13%	0%	97.91%
	99.5%	0.185676	4	0	0.23%	0%	99.76%
	99.9%	0.263154	0	0	0.00%	0%	100.00%
Vibration	95.0%	0.000000	19	0	5.35%	0%	94.88%
	97.5%	0.186088	7	0	1.97%	0%	98.11%
	99.5%	0.368756	6	0	1.69%	0%	98.38%
	99.9%	0.439512	4	0	1.13%	0%	98.92%

^1^ False positive rate is rounded to two decimal places. ^2^ False negative rate is rounded to two decimal places.

**Table 5:** Robustness of DSRF under different train–validation–test ratios and random data splits.

Type	Split	Detection Rate (Mean ± Std)	FPR (Mean ± Std)	Accuracy (Mean ± Std)
Current	60:20:20	100.00 ± 0.00%	0.49 ± 0.34%	99.52 ± 0.34%
	65:15:20	100.00 ± 0.00%	0.67 ± 0.43%	99.34 ± 0.42%
	70:10:20	100.00 ± 0.00%	0.64 ± 0.47%	99.37 ± 0.46%
Vibration	60:20:20	100.00 ± 0.00%	1.13 ± 0.80%	98.92 ± 0.76%
	65:15:20	100.00 ± 0.00%	1.41 ± 0.40%	98.65 ± 0.38%
	70:10:20	100.00 ± 0.00%	1.55 ± 0.20%	98.52 ± 0.19%

**Table 6:** Numbers of normal training and validation samples for current and vibration datasets.

Type	Total Normal Samples	Training Samples	Validation Samples	Test Samples
Current	8202	4291	1640	1641
Vibration	1774	1064	355	355

**Table 7:** Comparison of anomaly detection performance of PCA reconstruction, autoencoder, and DSRF for current and vibration datasets using 99.5th-percentile anomaly threshold.

Type	Method	TN	FP	TP	FN	Accuracy
Current	PCA	1638	3	32	0	99.82%
	Autoencoder	1637	4	32	0	99.76%
	DSRF	1637	4	32	0	99.76%
Vibration	PCA	349	6	16	0	99.38%
	Autoencoder	349	6	16	0	99.38%
	DSRF	349	6	16	0	99.38%

**Table 8:** Comparison of anomaly detection performance of PCA reconstruction, autoencoder, and DSRF for current and vibration datasets using 99.5th-percentile anomaly threshold under 60:20:20 training, validation, and test splits.

Type	Method	MATR (Mean ± Std)	AUROC (Mean ± Std)	Average Precision (Mean ± Std)
Current	PCA	1.631 ± 0.023	0.999730 ± 0.000334	0.9600 ± 0.0490
	Autoencoder	1.785 ± 0.054	0.999707 ± 0.000368	0.9585 ± 0.0510
	DSRF	3.159 ± 0.140	0.999733 ± 0.000329	0.9602 ± 0.0487
Vibration	PCA	3.211 ± 1.642	0.999965 ± 0.000070	0.9993 ± 0.0015
	Autoencoder	3.325 ± 1.741	0.999930 ± 0.000086	0.9985 ± 0.0018
	DSRF	4.461 ± 3.103	0.999930 ± 0.000141	0.9985 ± 0.0030

**Table 9:** Comparison of Hoyer sparsity and top-k feature concentration for the PCA reconstruction residual, DSRF anomaly residual, and DSRF remaining reconstruction residual in the current and vibration datasets.

Type	Metric	PCA Reconstruction Residual	DSRF Anomaly Residual	DSRF Reconstruction Residual
Current	Hoyer sparsity	0.240	0.362	0.042
	Top-5 concentration	2.22%	3.22%	0.56%
	Top-10 concentration	4.10%	5.86%	1.12%
Vibration	Hoyer sparsity	0.281	0.397	0.025
	Top-5 concentration	5.87%	8.16%	1.05%
	Top-10 concentration	9.80%	13.53%	2.10%

## Data Availability

The data supporting the findings of this study are available from the corresponding author upon reasonable request.
